# Fe_0.79_Si_0.07_B_0.14_ metallic glass gaskets for high-pressure research beyond 1 Mbar

**DOI:** 10.1107/S1600577522007573

**Published:** 2022-08-19

**Authors:** Weiwei Dong, Konstantin Glazyrin, Saiana Khandarkhaeva, Timofey Fedotenko, Jozef Bednarčík, Eran Greenberg, Leonid Dubrovinsky, Natalia Dubrovinskaia, Hanns-Peter Liermann

**Affiliations:** a Deutsches Elektronen-Synchrotron DESY, Notkestr. 85, 22607 Hamburg, Germany; b Beijing Synchrotron Radiation Facility, Institute of High Energy Physics, Chinese Academy of Sciences, Beijing 100049, People’s Republic of China; c Material Physics and Technology at Extreme Conditions, Laboratory of Crystallography, University of Bayreuth, 95440 Bayreuth, Germany; d Department of Condensed Matter Physics, Institute of Physics, P. J. Šafárik University, Šrobárova 1014/2, Košice 041 54, Slovakia; e Applied Physics Division, Soreq NRC, Yavne 8180000, Israel; f Bayerisches Geoinstitut, University of Bayreuth, 95440 Bayreuth, Germany; g Department of Physics, Chemistry and Biology (IFM), Linköping University, SE-581 83 Linköping, Sweden; ESRF – The European Synchrotron, France

**Keywords:** diamond anvil cell (DAC), amorphous metal gasket, metallic glass, axial and radial high-pressure X-ray diffraction, signal-to-noise ratio

## Abstract

A gasket is an important constituent of a diamond anvil cell (DAC) assembly, responsible for the sample chamber stability at extreme conditions for X-ray diffraction studies. In this work, we studied the performance of gaskets made of metallic glass Fe_0.79_Si_0.07_B_0.14_ in a number of high-pressure X-ray diffraction (XRD) experiments in DACs equipped with conventional and toroidal-shape diamond anvils. The experiments were conducted in either axial or radial geometry with X-ray beams of micrometre to sub-micrometre size. We report that Fe_0.79_Si_0.07_B_0.14_ metallic glass gaskets offer a stable sample environment under compression exceeding 1 Mbar in all XRD experiments described here, even in those involving small-molecule gases (*e.g.* Ne, H_2_) used as pressure-transmitting media or in those with laser heating in a DAC. Our results emphasize the material’s importance for a great number of delicate experiments conducted under extreme conditions. They indicate that the application of Fe_0.79_Si_0.07_B_0.14_ metallic glass gaskets in XRD experiments for both axial and radial geometries substantially improves various aspects of megabar experiments and, in particular, the signal-to-noise ratio in comparison to that with conventional gaskets made of Re, W, steel or other crystalline metals.

## Abstract 2

The performance of Fe_0.79_Si_0.07_B_0.14_ metallic glass as gasket material is investigated in a number of high-pressure X-ray diffraction experiments beyond 1 Mbar using conventional diamond anvil cells (DACs) or toroidal DACs. The combination of high mechanical stability and the smooth scattering background of the amorphous metal enable challenging high-pressure experiments, and result in a better signal-to-noise ratio in challenging powder and single-crystal X-ray diffraction studies.

## 1. Introduction

New developments in the field of diamond anvil cell (DAC) technology have significantly increased the range of experimentally achievable pressures and temperatures providing new opportunities for high-pressure physics, chemistry, materials and planetary sciences (Dubrovinsky *et al.*, 2015 ▸; Loubeyre *et al.*, 2020 ▸; Ji *et al.*, 2019 ▸; Kono *et al.*, 2016 ▸; Petitgirard *et al.*, 2019 ▸). Modern large-scale facilities, such as synchrotron radiation sources and X-ray free-electron lasers (XFELs), enable the most sophisticated experiments at extreme conditions. The technical demands of the high-pressure research community, *i.e.* a sub-micrometre focal size with high photon flux, are gradually being addressed through the upgrade programs of the major synchrotron facilities transforming to diffraction-limited storage rings (DLSRs), such as ESRF-EBS (https://www.esrf.eu/about/upgrade), APS-U (https://www.aps.anl.gov/APS-Upgrade) and PETRA IV (https://www.desy.de/research/facilities_projects/petra_iv/). In parallel, the high-pressure community contributes to the development, *e.g.* by improving the signal-to-noise ratio (SNR) through the improvement of the DAC assembly.

**Figure 1:**
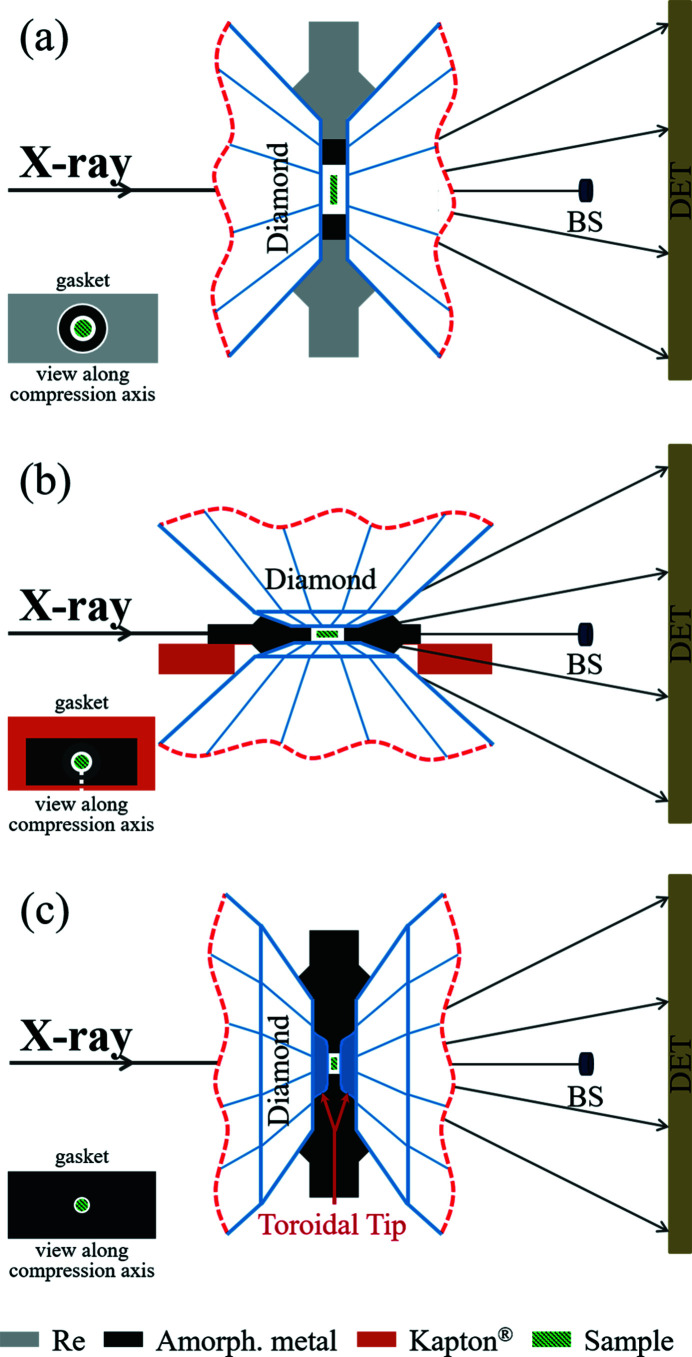
Schematic drawings of high-pressure XRD experiments in DACs with different geometries. (*a*) Axial geometry adopted for FeBO_3_ scXRD with Ne as PTM and for FeH pXRD with H_2_ as PTM. (*b*) Radial geometry employed for laser-heated GeO_2_ pXRD in a bevel DAC without PTM. (*c*) Axial geometry for the t-DAC experiment (ultrahigh-pressure pXRD experiments on Fe with MgO and Au with Ne). Inserts in the lower left corners indicate the gasket assembly as viewed along the DAC compression axis: the sample chamber is shown as a white circle; the white dashed line shown in the insert (*b*) indicates a slot in the Kapton foil, making it easier to remove Kapton after the gasket fixation by the diamond anvils. The materials of the gasket assembly and the sample are colour coded as shown at the bottom: light grey, dark grey, orange and green correspond to Re, Fe_0.79_Si_0.07_B_0.14_ metallic glass, Kapton and the sample, respectively. BS is the beam stop and DET indicates the position of a detector.

Among numerous aspects related to the design of the DAC assembly, the choice of a gasket material takes a central part. The gasket, containing a sample chamber, should ideally consist of a material with high yield strength and good ductility to support the contents of sample chambers up to target pressures, which often exceed 1 Mbar. For this reason, gaskets are often made of strong metals such as Re, W or steel. However, these crystalline materials made from high-*Z* elements produce intense X-ray diffraction signal even if only the strongly reduced intensity of the focused beam ‘tail’ interacts and scatters from the gasket material. Thus, most conventional gasket materials may significantly contribute to undesirable background scattering (on top of the Compton and diffuse scattering from the diamond anvils, *etc*.). These well known contributions complicate the diffraction pattern analysis and lead to a degradation of the SNR. For high-pressure experiments with axial geometry, where the X-ray beam goes through the diamond anvils parallel to the DAC’s compression axis [Fig. 1 ▸(*a*)], the ‘parasitic’ diffraction originating from the gasket material can be eliminated or considerably reduced, if the size of the X-ray spot is significantly smaller than the diameter of the sample chamber. In the case of radial geometry, where the X-ray beam is perpendicular to the DAC’s compression axis and X-rays go through the gasket [Fig. 1 ▸(*b*)], the diffraction from the gasket material is unavoidable. To reduce its contribution in the radial diffraction geometry, beryllium and ep­oxy mixtures containing low-*Z* elements or compounds are most commonly used. However, the properties of such materials may limit their application. For example, beryllium is toxic, and epoxies are often not hard enough. Among hard materials, gaskets made of diamond (Zou *et al.*, 2001 ▸), c-BN (Funamori & Sato, 2008 ▸; Wang *et al.*, 2011 ▸) and amorphous boron epoxies (a-BE) (Lin *et al.*, 2003 ▸; Merkel & Yagi, 2005 ▸; Rosa *et al.*, 2016 ▸) have been efficiently used. While such light-element materials are very transparent for high-energy X-rays, their sharp diffraction peaks produced by crystalline components of the ep­oxy mixtures (even diamond or c-BN have a large scattering volume) can significantly overlap with the signal from the sample causing a deterioration of the data quality. Therefore, despite the overall great progress in the application of various gasket materials to extreme conditions science, the optimization of the gasket performance within specific experimental constrains remains one of the most important challenges. The search for the optimal gasket material is always a compromise among the strength, X-ray transparency, chemical inertness, penetrability for gaseous pressure-transmitting media (PTM), simplicity for machining, costs and other factors of the material. This study is focused on improvement of the gasket material performance for X-ray diffraction applications at conventional and ultrahigh-pressure conditions.

Ultrahigh-pressure experiments (those beyond 1 Mbar) always require more elaborate preparation and, at times, unconventional diamond anvils and matching gasket designs. It was shown that double-stage DACs (ds-DACs) with secondary anvils, which enable pressure multiplication due to a stepwise decrease of the size of the diamond tip, allow one to achieve pressures as high as 1 TPa (Dubrovinskaia *et al.*, 2016 ▸). Following a similar idea, so-called toroidal-DACs (t-DACs) were introduced (Dewaele *et al.*, 2018 ▸; Jenei *et al.*, 2018 ▸; McMahon, 2018 ▸). Culets of their anvils are modified using a focused ion beam (FIB). The resulting toroidal profile features a central tip with an effectively reduced anvil size of about 15–25 µm in diameter and a few micrometres (<∼5 µm) in height. Depending on the shape of the t-DAC tips, the size of the sample chamber is ∼3–7 µm in depth and ∼10–15 µm in diameter. Experiments using t-DACs are usually conducted in axial geometry [Fig. 1 ▸(*c*)]. Pressures of a few megabars (beyond 600 GPa) have been achieved in t-DACs experiments. Ultrahigh-pressure experiments set a challenge for X-ray diffraction in terms of SNR, as the volume of the sample chamber and the amount of sample surrounded by gasket material are significantly reduced. We consider that introducing mechanically strong amorphous metal material with optimized yield strength and ductility will be of great benefit for a number of X-ray diffraction experiments, in particular it will benefit studies at pressures exceeding a megabar.

Amorphous metals (metallic glasses) are non-crystalline materials with a glass-like structure. They do not produce a background in the form of sharp diffraction peaks as crystalline materials do, and still possess high mechanical strength. Their properties highlight them as potential candidates for very efficient gasket materials. Gaskets made of metallic glass with the chemical composition Pd_40_Ni_40_P_20_ were successfully used in static high-pressure X-ray diffraction (XRD) experiments beyond 1 Mbar (He *et al.*, 2003 ▸). Another amorphous metal, Fe_0.79_Si_0.07_B_0.14_, with a significantly lower average number of electrons per atom, was recently used in dynamic high-pressure experiments and showed sufficient stability above 1 Mbar and temperatures >1000 K (Méndez *et al.*, 2020 ▸). In the present work, we extend the test of Fe_0.79_Si_0.07_B_0.14_ metallic glass performance to several challenging high-pressure experiments including single-crystal X-ray diffraction (scXRD) and powder X-ray diffraction (pXRD), involving conventional DACs and t-DACs. We note that, similar to the applicability of conventional gasket materials (Re, W, *etc.*), the applicability of Fe_0.79_Si_0.07_B_0.14_ material could also be limited, depending on a specific experimental case. Below, we share our experience and show practical examples demonstrating advantages of using Fe_0.79_Si_0.07_B_0.14_ in several challenging experiments.

## 2. Experimental methods

Metallic glass with the chemical composition of Fe_0.79_Si_0.07_B_0.14_ was used to make gaskets or gasket inserts for conventional metallic gaskets. The crystalline alloy with a nominal composition of Fe_0.79_Si_0.07_B_0.14_ (at.%) was prepared by arc melting. In order to achieve a good homogeneity of the precursor it was re-molten three times. Fully amorphous ribbons were prepared by rapid solidification of the molten material using a well established process – the planar flow casting technique – implemented by a single-roller melt spinner. This process is described in a number of publications (*e.g.* Overshott, 1979 ▸; Nair & Priyadarshini, 2016 ▸, *etc*.). Amorphous metal strips were cut from a melt-spun ribbon with a thickness of 23–25 µm produced at Institute of Physics, P. J. Šafárik University, Slovakia. The chemical composition of the metallic glass was confirmed by the electron microprobe analysis conducted at Bayerisches Geoinstitut (BGI, Bayreuth, Germany), providing a well consistent result of Fe_0.78(6)_Si_0.06(2)_B_0.15(7)_. The mechanical properties of Fe_0.79_Si_0.07_B_0.14_ should be remarkably close to those reported elsewhere (Luborsky & Walter, 1980 ▸; Hagiwara *et al.*, 1982 ▸; Nagarajan *et al.*, 1988 ▸; Sypień & Kusiński, 2006 ▸; Cadogan *et al.*, 2014 ▸) for the Fe–Si–B amorphous alloys of similar composition. For the sake of completeness, we present mechanical properties of a similar metallic glass with nominal composition of Fe_0.79_Si_0.05_B_0.16_ which can be found under the product code FE82-FL-000150 within the portfolio of GoodFellow Inc. Information on the mechanical properties of FE82-FL-000150 is listed in Appendix *A* [App appa].

Considering that thermal history and details of gasket preparation may influence the final properties of the material, we also conducted a study investigating the hardness of the 25 µm-thick Fe_0.79_Si_0.07_B_0.14_ foil. The first experiment employed a Shimadzu HMV-2 micro-hardness system with a micro-Vickers indenter (Tokyo Diamond Tools Mfg, model 17534). Loads of 0.98–19.61 N were applied for 5 s. A microscope with manual markers was used to measure the diagonals of the square indentation. Hardness measurements on thin foils are more complex in comparison with hardness measurements on bulk material due to the limitations on the maximal load (depth of indentation) and the thickness of the foils. Thus, this study was comparative and we used as a reference a Re foil of the same thickness (Alfa Aesar, 10307, 99.97% metal basis). We found that the observable hardness of Fe_0.79_Si_0.07_B_0.14_ is at least twice that of Re of the same thickness. Comparing our hardness measurements with the information shown in Appendix *A* [App appa], we find good agreement with the information available for elemental Re and FE82-FL-000150.

A more precise and accurate result on the hardness was obtained using a Nanoindenter G200 platform (KLA-Tencor, Milpitas, CA, USA), equipped with a Berkovich diamond tip (Synton MDP, Nidau, Switzerland). We used the continuous stiffness-based method (CSM) (Oliver & Pharr, 1992 ▸) in our quantitative analysis. The metallic foil was polished at the University of Bayreuth and indented at several different locations separated by a distance of at least 10 µm, so that their plastic zones did not overlap. For each measurement, loading was performed at a constant strain-rate of 0.025 s^−1^ up to a maximal indentation depth of at least 400 nm. We obtained a Vickers hardness of 6700 (400) MPa. Although the value is below the hardness of FE82-FL-000150, it is high and, as we demonstrate below, Fe_0.79_Si_0.07_B_0.14_ can enable a great number of demanding experiments.

In order to produce sample chambers in the gasket material, we used an electric discharge drilling machine (EDM) and a nanosecond pulse Excimer laser drilling machine operating at 193 nm (Optec^TM^ LaserShot Master). We noticed that the laser drilling may potentially cause recrystallization of glass at the circumference of the sample chamber hole, depending on the drilling parameters. That was never the case for EDM drilling. The recrystallization can be reduced or avoided by optimizing laser operation parameters, *e.g.* drilling at a low repetition rate (25–50 Hz, 6–7 mJ per pulse). Despite the minor presence of recrystallized gasket material, experiments with t-DACs showed that its contribution to the sample diffraction signal is indeed negligible.

All high-pressure XRD experiments in this work were performed at the P02.2 (ECB) beamline at PETRA III, DESY, Hamburg, Germany (Liermann *et al.*, 2015 ▸). The instrumental parameters were varied according to experimental conditions as discussed below. The data were collected using a Perkin Elmer XRD 1621 flat-panel detector. For the purpose of the data processing, we used various software packages including *CrysAlis Pro* (Rigaku), *Olex2* (Dolomanov *et al.*, 2009 ▸) in combination with *SHELXT* (Sheldrick, 2008 ▸, 2015*a* ▸,*b* ▸), *JANA2006* (Petříček *et al.*, 2014 ▸), *GSAS-II* (Toby & Von Dreele, 2013 ▸) and *DIOPTAS* (Prescher & Prakapenka, 2015 ▸).

The DAC experiments were divided into several cases (Table 1 ▸). In case A, a conventional Re gasket with an amorphous metal insert was used in the DAC experiments employing axial diffraction geometry [Fig. 1 ▸(*a*)]. Here we refer to the examples of single-crystal X-ray diffraction (scXRD, case A1) and powder X-ray diffraction (pXRD, case A2). In case B, pXRD experiments were conducted in radial geometry in a DAC supplied with a metallic glass gasket placed on a piece of Kapton foil [Fig. 1 ▸(*b*)]. Case C describes two applications of the metallic glass gaskets in t-DACs for ultrahigh-pressure pXRD experiments in axial geometry [Fig. 1 ▸(*c*)]. In case C1 we compressed Fe together with MgO, while case C2 describes the compression of Au together with Ne. Table 1 ▸ summarizes the experiments including instrumental parameters. Details of the individual experiments and assembly procedures are discussed below.

### 2.1. Case A: conventional Re gasket featuring an amorphous metal insert for scXRD and pXRD experiments in axial geometry

The amorphous metal gasket for the scXRD experiment (case A1) was prepared as follows. Firstly, a 250 µm-thick Re foil was pre-indented with a pair of diamonds with culet size of 150 µm/300 µm, bevel at 8° (Boehler-Almax design), to a thickness of ∼26–28 µm (slightly thicker than the metallic glass ribbon). Then, a hole of ∼110–120 µm in diameter was drilled in the centre of the indentation using EDM. A disc of a metallic glass was inserted into the hole and fixed within the primary Re gasket by a gentle compression of the latter between the anvils. Next, a central hole of diameter ∼75 µm (serving as the sample chamber) was drilled in the Fe_0.79_Si_0.07_B_0.14_ insert using EDM. The preparation process described above is somewhat flexible and the sequence of preparation steps can be adjusted. For example, one can first drill a hole in a metallic glass disc and then insert it into the hole of the Re gasket as was shown in the previous study by Méndez *et al.* (2020 ▸).

The pXRD experiment (case A2) was conducted in the pressure range up to 39 GPa by using a pair of 300 µm culet diamond anvils. A Re gasket was first indented to a thickness of 50–55 µm and then a 250 µm-diameter hole was drilled by EDM. In addition, we cut two disks of amorphous metal (23–25 µm thick) with similar outer diameter and loaded them as a stack, one on top of the other, into the Re gasket hole installed on a diamond anvil. The discs were fixed to the primary Re gasket by a gentle compression. Finally, the hole forming the sample chamber with a 150 µm inner diameter was drilled by EDM inside of the Fe_0.79_Si_0.07_B_0.14_.

### 2.2. Case B: metallic glass gasket placed on a piece of Kapton for pXRD experiment in radial geometry

Fig. 1 ▸(*b*) illustrates a schematic diagram of the radial pXRD experiment where the entire gasket was made from amorphous metal. The thickness of the as-produced metallic glass ribbon is thin enough (∼23–25 µm), so the process of gasket preparation can be simplified. To enable precise positioning of the tiny sample chamber on the tip of a diamond anvil we supplied the gasket with a support of a 125 µm-thick Kapton (DuPont^TM^) foil. The Kapton strip with a width of 3 mm was drilled using the Excimer laser drilling machine producing a hole of ∼400 µm in diameter. The hole served as a mounting aperture centred with the diamond of 150/300 µm, 8° bevel culet. Next, a metallic glass strip of rectangular shape (width below 1.4 mm) was cut from the metal glass ribbon using scissors and secured with instant glue above the hole in the Kapton foil. The dimensions of the amorphous glass strip can be potentially reduced without compromising the mechanical stability of the sample chamber keeping the sample at desired pressures. Shortly after that, a hole (concentric to the hole in the Kapton foil) with a diameter of 40 µm corresponding to the sample chamber was drilled in the metallic glass using the Excimer laser drilling machine.

A Kapton piece carrying the amorphous gasket can be easily attached to the diamond. During compression, the Kapton strip may bend or tilt. As a final step of the optimization, it can be fully removed from the cell assembly after the sample was loaded and compressed. This will reduce the Kapton contribution to the diffraction signal. In order to simplify the removal procedure, a slit can be cut into the Kapton strip as illustrated in the insert of Fig. 1 ▸(*b*).

A modified BX90 cell (Kantor *et al.*, 2012 ▸) dedicated for radial high-pressure XRD was employed in this test. The modification concept of BX90 comes from Lowell Miyagi. By modifying the piston and cylinder parts of the original BX90 design, two apertures perpendicular to the compression axis were expanded. This modification enabled data collection in radial geometry within a diffraction scattering cone of ∼54.5°. The cells were produced by Extreme Conditions Science Infrastructure of PETRA III, DESY.

### 2.3. Case C: metallic glass gasket in t-DACs for pXRD experiment in axial geometry

In our test we used a t-DAC in order to demonstrate the capability of the material under pressures of a few megabars. In case C1 we used Fe together with MgO, while in case C2 Au was loaded together with Ne PTM. The toroidal profiles of the diamond culets were prepared by using a focused ion beam instrument (FIB, SCIOS, FEI Thermofisher) installed at the DESY NanoLab (Stierle *et al.*, 2016 ▸) within the framework of the BMBF project No. 05K13WC3 (PI Natalia Dubrovinskaia). The milling bitmap is shown in Appendix *B* [App appb] (Fig. 10) along with an image of the resulting profile of the diamond culet, which is similar to the previous design suggested by Dewaele *et al.* (2018 ▸). After 4 h of milling with the Ga ion beam of 15 nA under 30 kV acceleration voltage, we can produce a single toroidal anvil with a conical shape tip of ∼4.3 µm in height and ∼20 µm in diameter. A piece of ∼25 µm-thick metallic glass ribbon with a hole of 40 µm was directly placed between the toroidal anvils for the first indentation. During the indentation, the diamond anvils were brought together and visible-light interferometry was used to measure the distance between the toroidal tips. At the moment of the hole collapse, if the distance was still larger than the target thickness of 4–5 µm, a new hole was made and the process of indentation was repeated. The process was stopped until the target thickness was achieved. The final sample chamber of ∼10 µm in diameter was drilled with several shots of the Excimer laser.

## 3. Results and discussions

### 3.1. Single-crystal X-ray diffraction of FeBO_3_ beyond 1 Mbar in a DAC with Ne PTM

The DAC assembly was prepared as described above for case A1; the experiment design is illustrated in Fig. 1 ▸(*a*). A single crystal of FeBO_3_ (Kotrbová *et al.*, 1985 ▸) was loaded into the sample chamber together with Ne as PTM and pressure marker (Fei *et al.*, 2007 ▸). The sample was compressed to ∼1.3 Mbar in this experiment. The scXRD data were collected during a rotation of the sample within ±32° around the ω-axis (perpendicular to the X-ray beam) with a step size of 0.5°. Additional technical information is summarized in Table 1 ▸.

**Figure 2:**
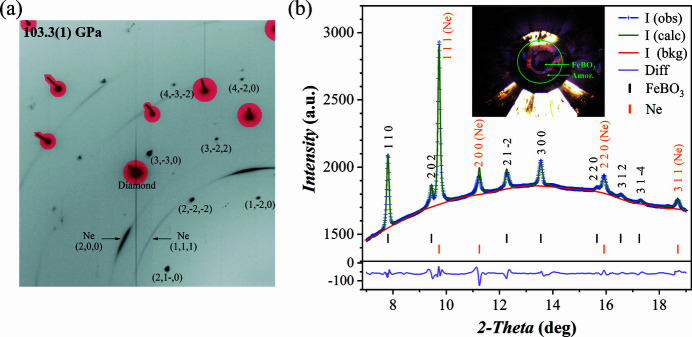
Diffraction patterns of FeBO_3_ single crystal in a DAC with Ne PTM at 103.3 (1) GPa. (*a*) A representative section of a 2D diffraction pattern. The Bragg peaks from diamond anvils are shaded in red; representative peaks of FeBO_3_ and Ne are indexed. (*b*) 1D diffraction pattern. The inset shows a micrograph of the sample chamber indicating the position of the FeBO_3_ single crystal and the inner edge of the Re gasket (green circle) which is also the outer edge of the amorphous metal insert; for scale, the diamond culet diameter is 150 µm. No strong scattering from the gasket material was observed at 100 GPa, although the X-ray beam was focused to 8 µm × 3 µm and the beam tails were non-negligible.

The collected XRD data are of excellent quality despite the megabar conditions and the occurrence of a spin-state transition at ∼50–54 GPa (Gavriliuk *et al.*, 2002 ▸; Vasiukov *et al.*, 2017 ▸). Fig. 2 ▸(*a*) shows a section of the 2D diffraction pattern at 103.3 (1) GPa with reflections of FeBO_3_ (single spots), Ne PTM, and diamond anvil Bragg reflections (masked in red) labelled. The contribution of gasket material to the detected signal is negligible despite the relatively large X-ray beam size used in this experiment (see Table 1 ▸). If the gasket would have been made entirely of Re or W, the diffraction lines of these strongly scattering materials would overlap with the diffraction spots of the single crystal substantially reducing the quality of the data. Here we focus on a single pressure point of 103.3 (1) GPa as the full data have been recently published (Xu *et al.*, 2022 ▸).

Indexing of the scXRD using *CrysAlis Pro* software confirmed the trigonal space group for FeBO_3_ at 103.3 (1) GPa. The full crystallographic information is provided in Table 2 ▸. The supporting information contains the corresponding CIF file. Fig. 2 ▸(*b*) shows the integrated 1D diffraction pattern and the Le Bail fit result performed by the *GSAS-II* software package. The Le Bail refinement converged to the unit-cell parameters of *a* = 4.2555 (8) Å, *c* = 11.796 (7) Å, which is in good agreement with the structure solution and refinement results based on scXRD (see Table 2 ▸).

### 3.2. Powder X-ray diffraction of FeH at 39 GPa in a DAC with H_2_ PTM

**Figure 3:**
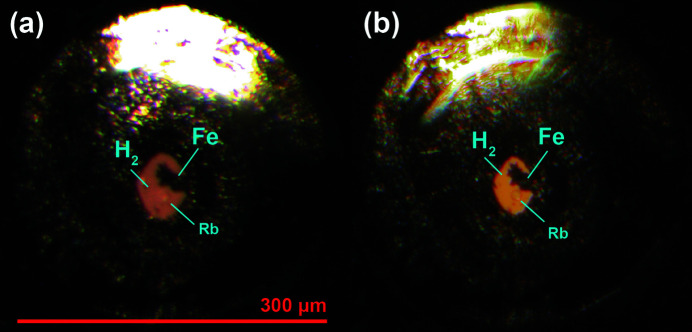
Micrographs of the sample chamber with ^57^Fe and ruby in H_2_ PTM: (*a*) at 2–3 GPa; (*b*) at 39 GPa after three weeks of equilibration.

**Figure 4:**
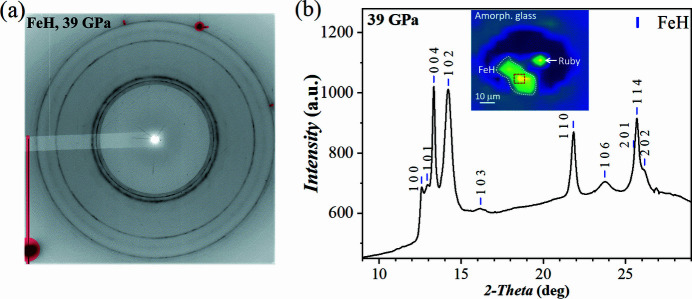
Diffraction pattern of FeH at 39 GPa with H_2_ as PTM loaded into the amorphous metal insert in a Re gasket. (*a*) 2D diffraction pattern without any evident contribution from the gasket material. The hydrogen signal is barely visible due to its low scattering power. Diamond Bragg peaks are masked with red markers. (*b*) The corresponding integrated 1D diffraction pattern including the indexing of FeH peaks. The insert shows the reconstruction of the phase distribution obtained from the analysis of the 2D pXRD map produced by sample scanning. We indicate the position of FeH and ruby in the sample chamber surrounded by amorphous metal. The boundary of the amorphous metallic material can be clearly seen.

Fig. 3 ▸ demonstrates the microp hotographs of the FeH loading. We performed XRD mapping over the entire sample chamber (see Table 1 ▸ for technical details). The representative 2D and 1D diffraction patterns are shown in Fig. 4 ▸. It is well known that Fe starts to react with H_2_ at very low pressures forming iron hydride, FeH (Hirao *et al.*, 2004 ▸). This reaction was confirmed in our pXRD data. The unit-cell parameters of FeH were determined by Le Bail refinement using *GSAS-II* resulting in *a* = 2.556 (1) Å, *c* = 8.33 (1) Å (space group *P*6_3_/*mmc*). It is almost impossible to detect H_2_ in the diffraction data [as illustrated in Fig. 4 ▸(*a*)] due to several effects: the low scattering power of H_2_, large scattering contrast between Fe and H (short accumulation times) and, potentially, the well known effect of H_2_ recrystallization in the form of larger single-crystal-like grains rather than a fine-grain powder upon slow compression. Additional analysis of Raman spectra, measured separately, confirmed the presence of the H_2_ vibron (Goncharov *et al.*, 2011 ▸). At the boundary of the sample chamber, the XRD map did not indicate formation of any Fe-, B- or Si-bearing polycrystalline compounds (*e.g.* hydrogen-containing) or recrystallized metallic glass. Our observations confirm that H_2_ should diffuse much less through Fe_0.79_Si_0.07_B_0.14_ in comparison with W, Re or steel, which form hydrides. The performance of this amorphous metal with other highly permeable gaseous PTMs, *e.g.* He, should be investigated additionally, but the results on H_2_ and Ne are very promising.

### 3.3. Radial powder X-ray diffraction of laser heating GeO_2_ at >1 Mbar in a DAC with no PTM

The DAC assembly was prepared as described above for case B. The experiment setup is illustrated in Fig. 1 ▸(*b*), and we refer to Table 1 ▸ for additional experimental details. We loaded a mixture of GeO_2_ powder (Sigma Aldrich 483702, ≥99.99% trace metals basis) and a small amount of Pt powder, serving as laser light absorber, into the sample chamber. The sample was compressed to >80 GPa and then laser heated at above 1000 K at two facilities (at BGI with pulsed laser, and at P02.2 with continuous laser). The sample was further compressed to about 90 GPa. The pressure was estimated from the diamond-Raman shift (Dubrovinskaia *et al.*, 2010 ▸). Repeated laser heating of the sample even up to ∼2000 K in the vicinity of 90 GPa did not cause any detectable damage to neither the metallic glass gasket nor to the diamonds. After heating at ∼90 GPa, the sample was further compressed at ambient temperature to pressures exceeding 1 Mbar.

**Figure 5:**
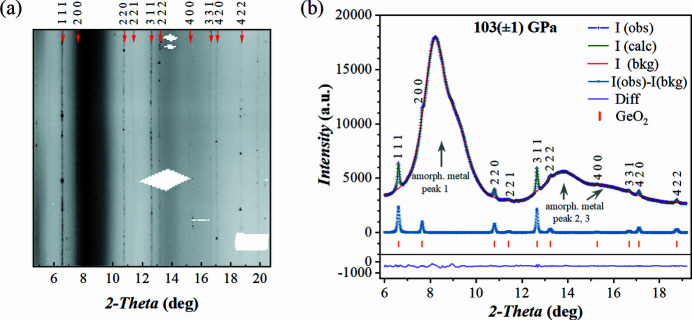
Radial diffraction patterns of GeO_2_ polymorph at 103 (1) GPa. (*a*) Unwrapped 2D diffraction pattern (the vertical axis corresponds to the azimuthal angle). Diffraction lines of GeO_2_ are indexed. The narrow and straight diffraction lines indicate small grains and low amount of strain in the sample. Diamond peaks were masked prior to 1D pattern integration. (*b*) The matching 1D diffraction pattern and the corresponding *GSAS-II* fit. The raw intensities, *I* (obs), are shown by the dark blue line. The light blue line corresponds to the raw data with the background intensity, *I* (bkg), subtracted. Tick marks indicate positions of the diffraction lines from GeO_2_ at 103 (1) GPa. *I* (calc) and Diff correspond to the calculated intensities (green line) and the residuals of the Le Bail fit (purple line), respectively.

Fig. 5 ▸ shows the 2D and 1D pXRD patterns collected at 103 (1) GPa in radial geometry. They are dominated by a few intense but relatively broad peaks originating from the amorphous metal and several sharp diffraction lines attributed to the pyrite-structured polymorph of GeO_2_. The amount of Pt in the cell was rather small, so that Pt peaks are not visible in the diffraction pattern. Although the scattering from the metallic glass gasket in radial geometry is omni­present [Fig. 5 ▸(*b*)], it does not prevent a reliable data analysis when it manifests as a smooth background. Indeed, it is very easy to fit the background profile and subtract it from the raw 1D diffraction pattern [as shown in Fig. 5 ▸(*b*)], which offers one of the biggest advantages in comparison with the conventional crystalline gasket materials (*e.g.* Be). The data were processed using the *GSAS-II* software package. The unit-cell parameter of pyrite-GeO_2_ (space group: 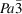 ) was determined to be *a* = 4.3591 (3) Å. As we mentioned earlier, the contribution of the amorphous gasket scattering signal can be further reduced by optimization of the amorphous gasket dimensions. It is also important to mention that we did not observe any additional contribution to the signal from potentially recrystallized metallic glass after repetitive laser heating of the GeO_2_ sample to 1000–2000 K, although we produced the pyrite-GeO_2_ in the entire area of the sample chamber.

### 3.4. Powder X-ray diffraction of Fe + MgO at multi-megabar pressure in a t-DAC

The DAC assembly was prepared as previously described for the case C. See also Fig. 1 ▸(*c*) and Table 1 ▸ for additional experimental details. We will start our discussion with case C1 focusing on the compression of metallic Fe together with MgO used as PTM.

**Figure 6:**
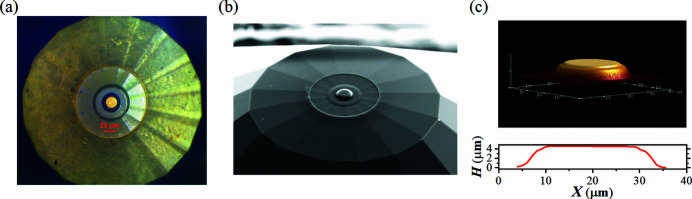
The toroidal diamond anvil fabricated from a standard diamond anvil (Almax-diamonds) with culet size of 40/300 µm, bevel angle of 8°. (*a*) Micrograph of the toroidal diamond anvil. (*b*) Photograph taken by electron beam in the FIB chamber with a sample stage inclination of 52°. (*c*) The tip of the toroidal anvil recorded by AFM.

Fig. 6 ▸(*a*) shows a micrograph of the primary diamond anvil with toroidal profile. A 3D image of the tip profile recorded by the atomic force microscope (AFM, Dimension Icon, Bruker) at the DESY Nanolab is shown in Fig. 6 ▸(*c*). The pressure chamber was loaded with a piece of iron powder (∼2–5 µm in diameter, Alfa Aesar 00170, 99.9+%) as sample material, and nano-crystalline powder of MgO (Sigma Aldrich 549649, particle size ≤50 nm) as pressure marker and PTM. Small grain size powders were selected in order to achieve a more uniform signal distribution along the Debye–Scherrer ring. While our Fe powder had larger grains in comparison with MgO, the transition of iron to the hexagonal closed-packed (h.c.p.) phase led to a finer grain powder signal with a more homogeneous distribution of intensity along the diffraction rings.

The sample chamber containing Fe and MgO was compressed with a step size of ∼30 GPa in the t-DAC up to 300 (5) GPa. Pressure was determined using the MgO equation of state (EoS) of Jacobsen *et al.* (2008 ▸) with *K*_0_ = 159.6 GPa and *K*′ = 3.74 (where *K*_0_ and *K*′ are the bulk modulus and it’s pressure derivative at 1 bar). If the MgO EoS of Zha *et al.* (2000 ▸) with parameters of *K*_0_ = 160.2 GPa and *K*′ = 4.03 is used, the highest stress condition experienced by MgO equates to 340 (5) GPa. Here we just show a snapshot from a larger work which will be published elsewhere.

**Figure 7:**
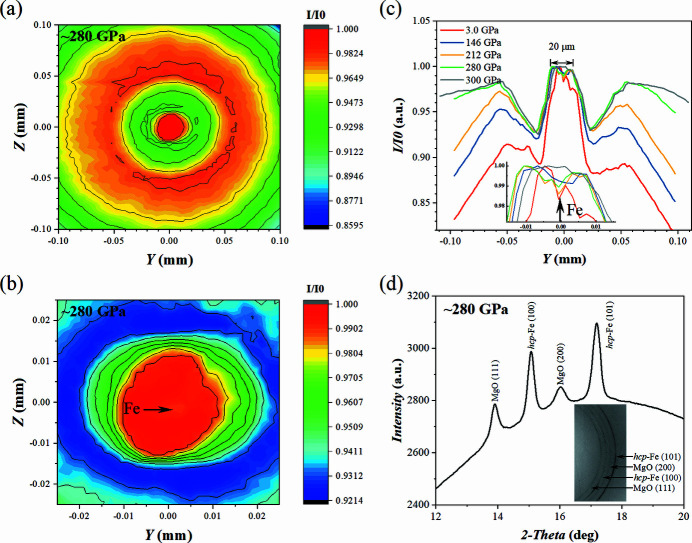
X-ray transmission maps (1D and 2D) across the anvils of the t-DAC and XRD patterns collected from the Fe + MgO sample. (*a*) The mapped area of 200 µm × 200 µm (step size of 4 µm) displays the transmission profile of the entire primary anvil culet which is increased to ∼105–110 µm after manufacturing of the toroidal tip. (*b*) The mapped area of 50 µm × 50 µm offers a detailed view (step size of 2 µm). The position of iron is indicated by the arrow and confirmed by 2D pXRD mapping. (*c*) 1D toroidal anvil X-ray transmission profiles at different pressures. (*d*) The integrated 1D diffraction pattern of Fe and MgO sample at ∼280 (5) GPa. The inset shows a part of a 2D diffraction image. The pressures here were calculated using the MgO EoS from Jacobsen *et al.* (2008 ▸). *I*/*I*_0_ corresponds to the normalized value of the X-ray intensity ratio of the transmitted and the incident beams. For the clarity of the representation, the value is normalized to the transmission intensity corresponding to the toroid sample chamber position.

At each compression step, the pressure chamber and its vicinity were scanned and 2D X-ray transmission maps were produced [Figs. 7 ▸(*a*)–7(*c*)]. The data were used to monitor the integrity of the pressure chamber and deformation of the diamond anvils. The 2D XRD patterns were also obtained in the scanning region and the data corresponding to 280 (5) GPa are shown in Appendix *B* [App appb] (Fig. 11). The corresponding integrated 1D XRD pattern is shown in Fig. 7 ▸(*d*) together with the representative 2D pattern. As shown in Appendix *B* [App appb] Fig. 11, a few reflections of h.c.p.-Fe and two weaker reflections of MgO are recorded. However, our observations indicate that a finite contribution of preferred orientation of MgO particles developed during the compression. The data are of high quality even at the highest-pressure conditions: sample signal intensity is strong, and the SNR is reasonably improved in comparison with conventional ultrahigh-pressure experiments involving polycrystalline Re or W gaskets. The lattice parameters were found to be *a* = 2.114 (2) Å, *c* = 3.378 (3) Å for h.c.p.-Fe and *a* = 3.459 (1) Å for MgO.

The experiment was conducted under non-hydro­static conditions. Compression in t-DAC generates enormous stresses and strains which are partially responsible for the visible peak broadening [Fig. 7 ▸(*d*)]. The non-hydro­static conditions may also be a reason for a large difference in pressures calculated using either MgO EoSes (Jacobsen *et al.*, 2008 ▸; Zha *et al.*, 2000 ▸) or Fe EoSes (Dewaele *et al.*, 2006 ▸). Considering the data shown in Fig. 7 ▸(*d*), the Fe EoSes estimate the pressure to be ∼338 and 347 GPa [according to Vinet and the third-order Birch–Murnaghan EoS of Dewaele *et al.* (2006 ▸), respectively]. These values are larger than those determined using MgO EoSes (Jacobsen *et al.*, 2008 ▸, Zha *et al.*, 2000 ▸). Our observations should stimulate the community attention with respect to the challenges of multiphase compression and pressure determination at non-thermally equilibrated conditions in conventional DACs in general (*e.g.* Glazyrin *et al.*, 2016 ▸), and in t-DACs in particular.

In our t-DAC experiments we used an X-ray energy of 25.6 keV. The accessible *Q*-range in the t-DAC at this energy range is quite limited. It may present a challenge in interpretation of the XRD data for a great number of inorganic materials, including simple solids under pressures exceeding 300 GPa. Access to X-ray sources with higher energy (allowing larger *Q*-range), higher flux (allowing stronger signal from smaller scattering volume), as well as smaller beam at the focal spot (allowing more precise mapping of the stress and strain state of the sample and suppressing ‘parasitic’ scattering from the gasket material) would be beneficial to t-DAC experiments at multi-megabar pressures. Our experiments are in line with the community requests towards the newer-generation X-ray sources such as DLSRs and XFELs. Both are considered the next step for exploration at extreme conditions, and, considering that the current generation of XFELs are impressive instruments offering superior time domain resolution, but operating at energies up to 25 keV, it becomes clear that only a combination of XFEL and synchrotron radiation facilities will enable the thorough characterization of multi-megabar samples compressed in t-DACs, especially in situations where single-crystal diffraction analysis has to be involved (*e.g.* ‘cook-and-look’ experiments *etc*.).

### 3.5. Powder X-ray diffraction of Au + Ne at multi-megabar pressure in a t-DAC

**Figure 8:**
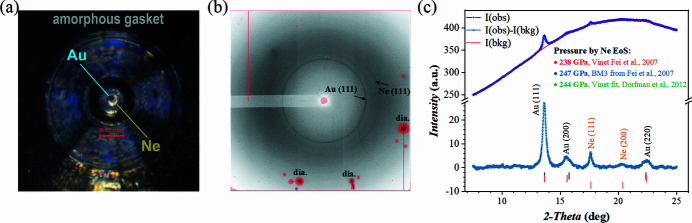
(*a*) Micrograph of the t-DAC assembly loaded with Au and Ne at ∼65 GPa. The darker area inside the sample chamber corresponds to Au powder, while the brighter area, illuminated by back light, corresponds to Ne. (*b*) 2D X-ray diffraction pattern collected at ∼247 GPa [pressure determined by the third-order Birch–Murnaghan EoS (BM3-EoS) of Ne from Fei *et al.* (2007 ▸)] with indication of (111) peaks of Au and Ne. Strong diamond peaks are indicated separately. (*c*) 1D diffraction pattern corresponding to (*b*). With respect to the X-ray background signal on the 1D pattern, we observe a continuous variation of intensity attributed to the contribution from Compton scattering of the diamond anvils and a negligible contribution from the amorphous gasket. The raw data corresponding to *I* (obs) are indicated with a dark blue line; the light blue line corresponds to the raw data with background intensity *I* (bkg) subtracted. Black/red and orange ticks shown under the 1D pattern indicate peak positions for Au and Ne, respectively. The black ticks correspond to Au *hkl* reflections with Le Bail fit, while the red ticks indicate diffraction peak positions of Au fitted individually. The pressure values were estimated by different Ne EoSes.

If we consider various aspects of quickly developing t-DAC techniques, we quickly come to an understanding that quasi­hydro­static pressure conditions as well as the precision of pressure determination are of great importance, but the topics are not well explored. Here, we extended our tests of t-DAC and present the case C2, where gold particles were loaded into a sample chamber of a t-DAC together with Ne as PTM, which are often used as pressure sensor materials in methodological studies. Additional details can be found in Table 1 ▸. Fig. 8 ▸(*a*) shows a micrograph of the t-DAC cell pre-loaded to ∼65 GPa confirmed by the diamond Raman peak measured at the tip of the toroid anvil. Great mechanical properties of the gasket material allowed us to compress Ne and Au clamped between the toroidal tips to above 2 Mbar. Figs. 8 ▸(*b*) and 8(*c*) present 2D and 1D diffraction patterns of Au and Ne at the highest pressure we achieved. Exceptionally simple backgrounds of the diffraction patterns [Figs. 8 ▸(*b*) and 8(*c*)] allowed unambiguous determination of the unit-cell volumes of Au (*V*_Au_) and Ne (*V*_Ne_) as a function of pressure. In this t-DAC experiment, we investigated cross correlations between *V*_Au_ and *V*_Ne_ and compared them with known EoSes. In Fig. 8 ▸(*c*), we show the pressure results calculated via different Ne EoSes (Dorfman *et al.*, 2012 ▸; Fei *et al.*, 2007 ▸). We also observed a small peak position shift of Au between the individual peak profile fitting and the Le Bail fitting results. As shown, the black and red tick marks in Fig. 8 ▸(*c*) correspond to Au *hkl* reflections by Le Bail fit and individual peak profile fitting, respectively. The small offset for Au (200) with respect to the ‘ideal’ position could be attributed to a small deviatoric stress. It is similar to the behaviour of Ne (200) at low and moderate pressures.

The small number of visible diffraction peaks and their low intensities does not allow us to perform a full deviatoric stress analysis for neither Au nor Ne and thus characterize the magnitude of non-hydro­static stresses and strains. But it is equally important to show clear experimental evidence indicating a deviatoric stress presence in t-DAC experiments, emphasizing a complicated picture of multiphase compression in the latter.

**Figure 9:**
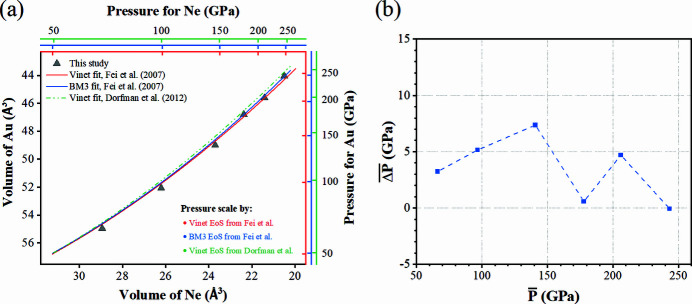
Data describing Ne + Au compressed in a t-DAC at room temperature in comparison with the literature (Fei *et al.*, 2007 ▸; Dorfman *et al.*, 2012 ▸). (*a*) Measured unit-cell volumes of Ne and Au are compared with EoSes from the literature. (*b*) Average pressure deviation between Au and Ne as a function of the average pressure calculated using the indicated EoSes. The former value is calculated as 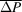 = 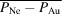 , while the latter is defined as 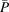 = 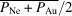 .

**Figure 10:**
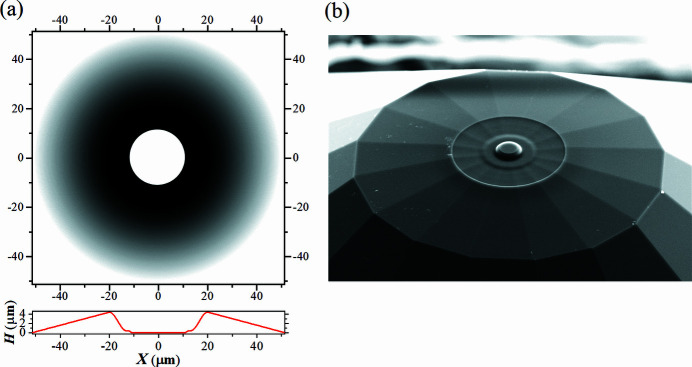
(*a*) Bitmap employed for the toroidal diamond anvil fabrication. Toroid modification was produced on top of a standard diamond (Almax-diamonds) with the initial culet size of 40/300 µm, bevel angle of 8°. The 1D milling profile is indicated. (*b*) Photograph made in FIB presenting a view of the milled diamond with a mounting stage inclination of 52°.

**Figure 11:**
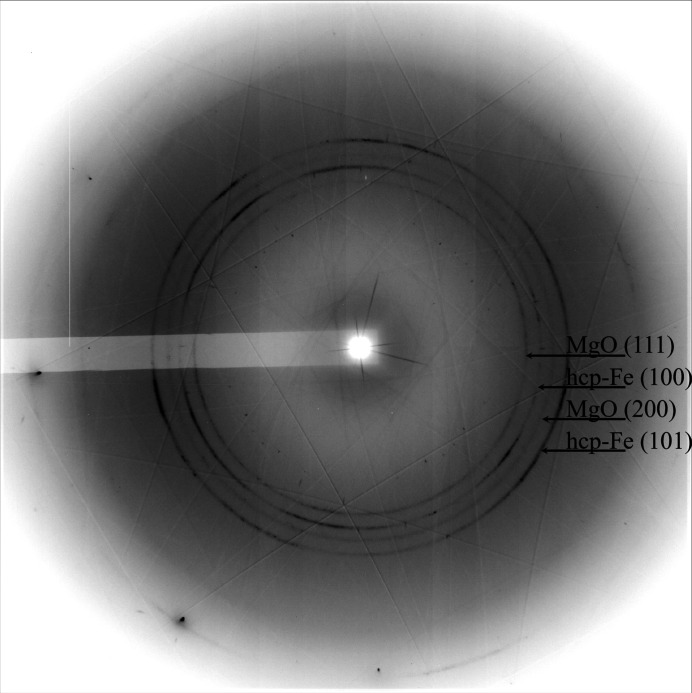
The full 2D diffraction pattern of Fe + MgO collected at ∼280 (5) GPa [MgO EoS (Jacobsen *et al.*, 2008 ▸)] or 313 (5) [MgO EoS (Zha *et al.*, 2000 ▸)] collected from the t-DAC. The sample chamber was surrounded by an Fe_0.79_Si_0.07_B_0.14_ metallic glass gasket. We observe a strong, clear signal with a negligible contribution from the gasket material. As indicated, the strongest powder-like signal belongs to h.c.p.-Fe and MgO. There is an additional contribution from diamond anvils in the form of strong spots. Data were collected at a wavelength of 0.4834 Å.

Fig. 9 ▸(*a*) shows the volume of Au (*V*_Au_) as a function of the volume of Ne (*V*_Ne_) upon compression in a t-DAC. Considering the literature data, in Fig. 9 ▸(*a*) we also present the calculated *V*_Au_ and *V*_Ne_ using some of the pressure scale studies reporting simultaneous Au and Ne compression (Fei *et al.*, 2007 ▸; Dorfman *et al.*, 2012 ▸). The right and top axes indicate pressure scales calculated from the EoSes with the corresponding labels shown in the figure. Although there is no perfect match, we could imagine better matching results when using the third-order Birch–Murnaghan EoS (BM3-EoS) from Fei *et al.* (2007 ▸). Small deviations are observed when comparing our data with the literature, *e.g.* Dorfmann *et al.* (2012 ▸). However, the latter publication shows a strong overlap of the diffraction peak from Au, Re and even NaCl, which could have led to interferences during data analysis. At pressures below 150 GPa [Fig. 9 ▸(*a*)] we see a stronger deviation of *V*_Au_ versus *V*_Ne_ from all indicated EOSes. Basically, for the same experimental conditions, *V*_Au_ was smaller than it should have been if we considered *V*_Ne_ as a reference for pressure at thermodynamic equilibrium conditions. This observation can be an indication of the stronger influence of deviatoric stresses at lower pressures, below 150 GPa in our case, which seem to become reduced toward higher pressures. The diffraction pattern shown in Fig. 8 ▸(*c*) represents a great example illustrating the advantages of amorphous metallic gasket or gasket insert (with respect to the background and sample signal overlapping issue) over the crystalline gaskets.

We supplement the discussion with Fig. 9 ▸(*b*), where we present an average pressure difference of 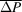 = 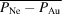 as a function of the whole average pressure 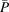 = 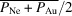 calculated from the EoSes shown in Fig. 9 ▸(*a*). The data indicate that during the compression process in the t-DAC loaded with Au and Ne we saw a pressure deviation below 5%. We consider this observation as important, especially given the tiny dimension of the t-DAC sample chamber and the conditions of great stress and strain. Our data inspire cautious optimism that, despite the small sample chamber volume, EoSes of Ne and Au measured at lower pressure ranges can be indeed applied to t-DAC loadings with quasi­hydro­static pressure media, and can be reasonably extrapolated to higher pressures [*e.g.* EoS of Fei *et al.* (2007 ▸)].

## 4. Conclusions

In this work we studied the performance of gaskets and gasket inserts made of Fe_0.79_Si_0.07_B_0.14_ metallic glass in several high-pressure single-crystal and powder X-ray diffraction experiments at pressures of ∼1 Mbar and above. Although the high-pressure/temperature stability of Fe_0.79_Si_0.07_B_0.14_ has not been investigated in detail so far, our results show that the material performs well and exhibits sufficient mechanical stability for the DAC assembly even at pressures as high as a few megabar and even when in contact with sample heated to ∼2000 K. The mechanical performance of Fe_0.79_Si_0.07_B_0.14_ is excellent even without the support of a thicker Re gasket, as shown in our example featuring Au and Ne compression in a t-DAC. We consider that implementation of Fe_0.79_Si_0.07_B_0.14_ metallic glass gasket paves the way for improving the SNR in X-ray diffraction work, which is indeed of great importance for ultrahigh-pressure experiments in t-DACs and dsDACs aiming for extremes, but also beneficial for experiments at lower pressures conducted with larger X-ray beams (*e.g.* combined X-ray imaging with diffraction).

Our tests confirmed that Fe_0.79_Si_0.07_B_0.14_ can efficiently hold conventional and exotic gaseous PTM, like Ne and H_2_. Sample laser heating or other high-temperature treatment [*e.g.* resistive heating by Méndez *et al.* (2020 ▸)] of the sample chamber during high-pressure experiments may cause partial recrystallization of the amorphous metal material, but we found that the contribution from the recrystallized material to the sample signal is negligible because of considerably lower scattering power (low *Z*) in comparison with conventional Re and W gaskets.

We anticipate a heated discussion with respect to Fe–Si–B material applications and limitations. As we show above, this material can be perfectly applied for a great number of studies, but it will all depend on the specific application. The above-reported material and similar materials, for example Ni_0.78_Si_0.08_B_0.14_ (NI80-FL-000150, GoodFellow), will enable various complicated multiprobe experiments, *e.g.* X-ray emission/absorption + XRD, Mössbauer spectroscopy + XRD, X-ray imaging + XRD, *etc*. Considering the specific example of Ni_0.78_Si_0.08_B_0.14_, it can be used in cases when additional X-ray spectroscopic signal from Fe is undesirable. The field of metallic glasses is extensive and there is still much to be learned and evaluated with respect to experimental high-pressure science.

On a final note, we studied the performance of a single Fe–Si–B amorphous metal composition (Fe_0.79_Si_0.07_B_0.14_), but the Fe–Si–B phase diagram offers a broad range of compounds. Many of those can be potentially quenched into an amorphous state. Therefore, we further refer to Miettinen *et al.* (2019 ▸) for additional information regarding other possible metallic glass materials within the Fe–Si–B family and their crystallization paths, which, along with Fe_0.79_Si_0.07_B_0.14_, could also potentially be used as novel amorphous gasket materials for application in diverse high-pressure high-temperature studies at third- and fourth-generation synchrotrons as well as at X-ray free-electron laser facilities.

## Supplementary Material

Crystal structure: contains datablock(s) global, I. DOI: 10.1107/S1600577522007573/ok5080sup1.cif

Structure factors: contains datablock(s) I. DOI: 10.1107/S1600577522007573/ok5080Isup2.hkl

## Figures and Tables

**Table 1:** Summary of the experimental information

Case	Sample, PTM	Diamond anvil type	X-ray beam: wavelength, beam size†	Gasket or gasket insert dimensions‡	Maximum sample pressure, temperature	
A1	FeBO_3_ (single crystal), Ne	Boehler-Almax, Ø150/300 µm, 8° bevel	0.2898 Å, 8 µm × 3 µm	Ø_Re_/Ø_A_ = 120/75 µm, *t* = 20 µm	∼1.3 Mbar, 300 K	
A2	FeH (sub-micrometre grain powder), H_2_	Boehler-Almax, Ø300 µm	0.4845 Å, 2 µm × 2 µm	Ø_Re_/Ø_A_ = 200/100 µm, *t* = 30 µm	∼0.4 Mbar, 300 K	
B	GeO_2_ (sub-micrometre grain powder), no PTM	Standard design, Ø150/300 µm, 8° bevel	0.2898 Å, 8 µm × 3 µm	Ø_K_/Ø_A_ = 400/40 µm, *t* = 25 µm	∼1.0 Mbar, below 2000 K	
C1	Fe (sub-micrometre grain powder), MgO	t-DAC, tip Ø20 µm, tip height ∼4.3 µm	0.4834 Å, 1.5 µm × 0.9 µm	Ø_A_ = 10 µm, *t* = 4 µm	∼3.0 Mbar, 300 K	
C2	Au (sub-micrometre grain powder), Ne	t-DAC, tip Ø20 µm, tip height ∼4.3 µm	0.4839 Å, 1.3 µm × 1.0 µm	Ø_A_ = 10 µm, *t* = 4–5 µm	∼2.5 Mbar, 300 K	

† Beam size at the focal spot corresponds to H × V at FWHM.

‡ Ø_Re_, Ø_A_ and Ø_K_ correspond to the diameters of holes inside the Re, metallic glass and Kapton pieces, respectively. *t* is the gasket thickness before sample loading.

**Table 2:** Crystallographic information for FeBO_3_ single crystal at 103.3 (1) GPa *R*(*F* ^2^), *wR*(*F* ^2^) are the crystallographic *R*-factors. *I* corresponds to intensity, while *F* is the atomic scattering factor. *N*_ref_ and *N*_Par_ are the number of unique reflections (merged) used in analysis and the number of refined parameters, respectively. *U*_iso_ is the isotropic thermal displacement parameter.

Crystal information			
Experiment wavelength (Å)		0.2898	
Space group, number		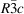 , 167	
*Z*		6	
*a* (Å), *c* (Å)		4.2510 (2), 11.772 (12)	
*V* (Å^3^)		184.2 (2)	

Refinement details			
*R*(*F* ^2^); *I* > 2σ(*I*)		5.8%	
*wR*(*F* ^2^); *I* > 2σ(*I*)		12.2%	
Completeness		24.6%	
*N*_ref_/*N*_par_		42/5	
*HKL* statistics		−8 ≤ *H* ≤ 8; −7 ≤ *K* ≤ 6; −4 ≤ *L* ≤ 5	

Structural parameters			
Atom	Site symmetry	Atomic coordinates (*x*, *y*, *z*)	*U*_iso_
Fe	6b	0, 0, 0	0.0037 (7)
O	18e	0.3094 (10), 0, 0.25	0.0050 (10)
B	6a	0, 0, 0.25	0.0110 (30)

**Table 3:** Mechanical properties of Fe–Si–B metallic glasses

Properties of FE82-FL-000150, GoodFellow Inc.	Value
Nominal composition	Fe_0.79_Si_0.05_B_0.16_
Hardness – Vickers (MPa)	8820
Tensile strength (MPa)	1500
Density (g cm^−3^)	7.28
Crystallization temperature (°C)	515
Curie temperature (°C)	405

## Appendix A Mechanical properties of Fe–Si–B metallic glasses

Table 3 ▸ shows the mechanical properties of Fe–Si–B metallic glasses. Considering Fe–Si–B amorphous metallic systems, we find a great number of producers of this inexpensive material. Our review of the materials based on Fe–Si–B and produced by different manufacturers, *e.g.* Hitachi 2605SA1, MAGNETICS MACC0004 (similar to Fe_0.75_Si_0.15_B_0.10_), shows that the Vickers hardness varies only slightly with substantial changes of B and Si content and typically exceeds 8000 MPa. We direct the reader to the current documentation of the manufacturers for additional details. Information on the Re and W hardness can be found in Samsonov (1968 ▸), *e.g.* Re foil has a Vickers hardness in the range 1350–7850 MPa while that of W ranges from 3430 to 4600 MPa. However, we emphasize that material properties depend on a great number of parameters, including thermal history, grain size, *etc*. The review of steels and their hardness goes beyond the scope of this work, but there are a great number of handbooks available that provide ample information on the physical properties, *e.g.* Nayar (2009 ▸).

## Appendix B Bitmap and fabrication results

Figs. 10 ▸ and 11 ▸ show the bitmap and fabrication result of the t-DAC and full 2D diffraction pattern of Fe + MgO in a t-DAC.
